# Understanding low vision

**Published:** 2012

**Authors:** Clare Gilbert

**Affiliations:** Co-director, International Centre for Eye Health, London School of Hygiene and Tropical Medicine, Keppel Street, London WC1E 7HT, UK; Clinical Advisor, Sightsavers.

**Figure:**
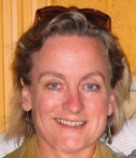
Clare Gilbert

## Who is likely to have low vision?

As a rule of thumb, the following people are likely to need low vision services and must be referred wherever possible:

- All children who have undergone bilateral cataract operations, both those with pseudophakia and those with aphakia
- People with diabetic macular oedema whose vision remains poor despite laser treatment
- People with age-related macular degeneration
- Children with oculocutaneous albinism
- People with optic atrophy, whatever the cause
- Any person who still has difficulty performing their daily activities because of their vision, even after treatment and refraction.

## What does low vision look like?

People with low vision are affected in different ways. They may suffer from some or all of the following:

- Severely reduced visual acuity
- Blurred vision
- Visual field loss: central or peripheral
- Loss of contrast sensitivity
- Increased light sensitivity.

**Figure 1.**
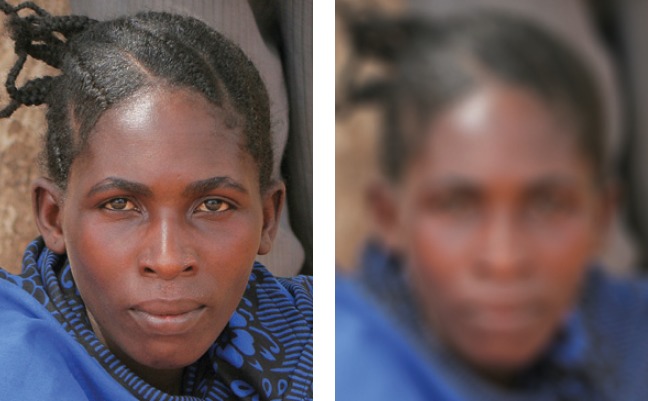
***Blurred vision***. People with blurred vision (right) have difficulty seeing details, both at distance and nearby; they often have problems with glare. Printed materials and colours might seem faded

Many people with low vision suffer from blurred vision (Figure 1), for example if they have scarring on their corneas.

**Figure 2.**
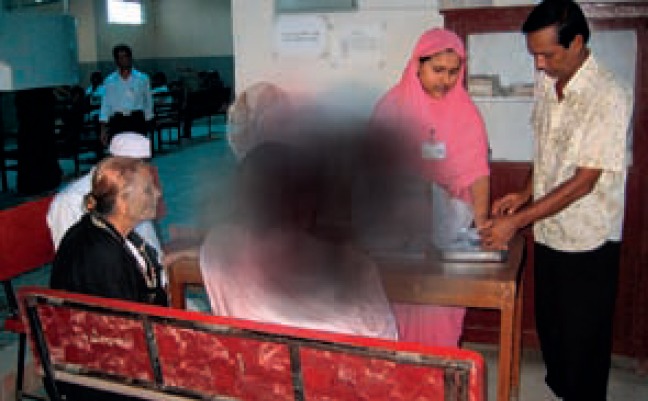
***Loss of central vision***. “Is the man sitting down my husband, and is there a seat for me?”

People with optic atrophy or age-related macular degeneration will have loss of central visual acuity (Figure 2), which means that tasks requiring good central vision will be difficult. For example, reading, writing, threading a needle and sewing, putting on make-up, recognising people, seeing where their food is on the plate and whether they have finished eating, seeing if their clothes are clean, finding their own pair of shoes. If they have a full field of peripheral vision then mobility will be less of a problem.

**Figure 3.**
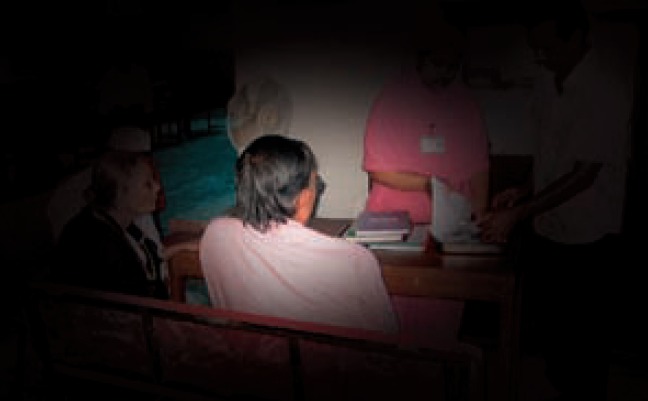
***Loss of peripheral vision***. “How many other people are there in the room?”

Someone with glaucoma or retinitis pigmentosa will have constricted visual fields, i.e. loss of peripheral vision (Figure 3). This makes it difficult to move around without bumping into objects on the floor. People may have difficulty finding things they have dropped. Reading may still be possible, but difficult.

**Figure 4.**
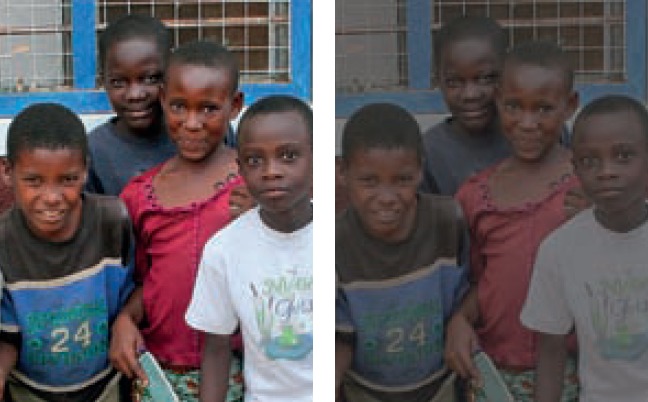
***Loss of contrast sensitivity***. With normal contrast sensitivity (left), it is easy to recognise faces. With reduced contrast sensitivity (right) this becomes more difficult

Loss of contrast sensitivity (Figure 4) can have a very big impact on someone's visual function, making it difficult to recognise faces or find food on a plate of similar colour.

**Figure 5.**
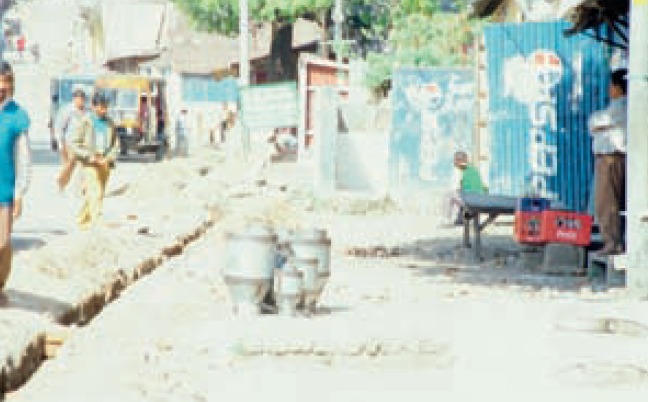
***Increased light sensitivity***. This is how a street scene in bright sunlight would look to someone who has increased light sensitivity

Increased light sensitivity makes it very difficult for people to see detail or make sense of what they see if they are in bright light, or glare (Figure 5).

